# Chronic supplementation of noni in diabetic type 1-STZ rats: effects on glycemic levels, kidney toxicity and exercise performance

**DOI:** 10.1186/s13098-023-01171-1

**Published:** 2023-10-04

**Authors:** Débora de Oliveira Fernandes, Fernanda Gracia César, Bruno Pereira Melo, Jéssica da Silva Faria Brandão, Kelvin Jaques dos Santos, Marcelo Teixeira de Andrade, Marisa Cristina da Fonseca Casteluber, Moisés Vieira de Carvalho, Luiz Alexandre Medrado de Barcellos, Danusa Dias Soares, Juliana Bohnen Guimarães

**Affiliations:** 1 State University of Minas Gerais – Ibirité Unit, Ibirité, Brazil; 2 Exercise Physiology Laboratory, School of Physical Education, Physiotherapy and Occupational Therapy, Federal University of Minas Gerais, Belo Horizonte, Minas Gerais Brazil; 3 Department of Science of Human Movement, State University of Minas Gerais - Ibirité Unit, Av. São Paulo, 3996, Vila do Rosário, Ibirité, 32400-000 Minas Gerais Brazil

**Keywords:** Exercise, fatigue, glucose, Oxygen consumption, Renal toxicity

## Abstract

Noni is a fruit with potential medicinal use preventing elevated blood glucose levels in diabetes mellitus. Its effects have been attributed to an antioxidant property in several other diseases. However, the effects of noni-chronic supplementation on exercise performance in the presence of diabetes conditions are not known. Thirty-two male Wistar rats were used to verify the effects of chronic noni (*Morinda citrifolia L*) juice administration on glycemia, triglyceride levels, and its relation to physical performance. In addition, it was verified if chronic noni supplementation is safe for clinical use through kidney morphology analysis. In half of the rats, diabetes mellitus (DM) was induced with STZ. All rats were submitted to an incremental workload running test (IWT) until fatigued so that oxygen consumption and performance indexes (exercise time to fatigue and workload) could be analyzed before noni administration. Then, the control and DM groups received a placebo (saline solution) or noni juice (dilution 2:1) at a dose of 2 mL/kg once a day for 60 days. The result was four groups: control + placebo (CP), control + noni (CN), DM + placebo (DMP), and DM + noni (DMN). Our dose was based on in previous study by Nayak et al. (2011) that observed a significant reduction in glycemia with 2 ml/kg of the noni juice without any toxicity effect cited. Groups were then given a third IWT to verify the effect of the noni juice on exercise performance (exercise time to fatigue, workload, maximal oxygen consumption) and glycemia. Twenty-four hours after the third test, all animals were euthanized and blood and kidneys were removed for posterior analysis. The DM induction with STZ impaired the performance by 39%. Noni administration improved the time to fatigue and workload in DM rats beyond reducing hyperglycemia. These results could be associated with an improved energy efficiency promoted by noni ingestion, since the oxygen consumption was not different between the groups, although the exercise was longer in animals with noni ingestion. Our results provided evidence that chronic noni administration causes kidney damage since increased Bowman’s space area in the control rats, suggesting glomerular hyperfiltration at the same magnitude as the non-treated DM group.

In conclusion, chronic noni ingestion promoted glycemic control and improved the performance in DM rats but caused kidney toxicity.

## Introduction

Diabetes mellitus (DM) is a chronic disease characterized by an absolute or relative deficiency of insulin or its action that promotes hyperglycemia [1, 2]. Controlling blood glucose levels within physiological parameters is fundamental to avoiding complications associated with DM. In general, such control is possible by the administration of exogenous insulin or oral drugs, which reduces hyperglycemia events [3].

In this context, the ingestion of certain fruits and herbs seems to contribute to glycemic control through a hypoglycemic effect in DM people. For example, *Morinda citrifolia L.* (Rubiaceace), popularly known as noni, is a fruit native to Polynesia, Asia, Australia, and the Brazilian coast and has been extensively associated with preventing elevated blood glucose levels in diabetics. Its medicinal use has been attributed to a potential antioxidant effect (i.e., ascorbic acid and flavonoids) for the treatment of wounds, infections, menstrual and intestinal irregularities, hypertension, and cancer [4]. Specifically, it has been shown that ingestion of fermented noni juice (10x the noni sample amount w/v) reduced blood glucose levels and increased the use of lipids in genetically obese type II diabetes mice [5]. Furthermore, 70% ethanolic extract noni administration in vitro muscle cell (C2C12) cultures induced an increase in adenosine 5’-monophosphate-activated protein kinase (AMPK) pathway activation and GLUT4 translocation with increased glucose uptake [5]. Furthermore, ingestion of fermented noni juice seems to regulate metabolism through the gene expression levels of CPT-1 and PPARα increased which contribute to reducing the level of lipids synthesis and promote fatty acid β-oxidation [6].

Some results have indicated that fermented noni juice promotes lower glycemic levels [5, 7], however, several studies are mentioning the medicinal purposes of using the fruit diluted with water [8, 9]. The way noni is prepared has produced controversial results [8, 10]. In addition, the period of the noni juice supplementation could interfere with these responses and produce toxicity effects. The study by Leal-Silva et al. (2023) verified toxic effects for the liver and reproductive development in the mother and fetal abnormality after ingestion of noni aqueous extract with a dose higher than 400 mg/kg for 21 days. However, in a lower dose of the noni ingestion, any toxicity effect was not verified [11]. Thus, studies are necessary for clinical confirmation of the fruit and verification of any deleterious effects.

In the field of sports science, some studies have shown a similarity between the mechanisms produced by noni administration and exercise, which promotes glucose uptake as a consequence of AMPK-enhanced GLUT4 translocation, which is insulin-independent [12]. Briefly, the contraction muscle activates the increasing of intracellular Ca^2^ and consequently, translocation of GLUT4 induced by kinase protein dependent on the CA-calmodulin mechanism [12]. In addition, exercise promotes increased activity of the glycolytic pathway and consequently also activates ATP phosphorylation-dependent kinases, such as AMPK, inducing transporter translocation GLUT4 [12]. This mechanism has also already been proposed after noni supplementation, suggesting increasing mitochondrial biogenesis, since there was an increase in UCP3 and PGC1ɑ in muscle [8].

Furthermore, it is suggested that the blood glucose level may influence performance during exercise. However, such a result would conflict with studies that the effects of increased glucose availability on performance showed anticipation [13], delay [14], or no effect on fatigue [15].

Therefore, this study aimed to evaluate the effect of chronic noni juice administration on blood glucose levels and its relation to the physical performance of DM rats. In addition, to determine if chronic intake of noni juice is toxic, an evaluation was conducted using renal analysis.

## Materials and methods

### Animals

Thirty-two male Wistar rats weighing about 200 g (6 weeks of age) at the beginning of the study were used. They were acquired from the Central Bioterium ICB/UFMG and habituated to a local bioterium over seven days. They were housed in collective polypropylene cages (four rats per cage) under controlled light (0500–1900 h) and temperature (23.5 ± 1.0 °C) conditions with water and rat chow provided ad libitum.

### DM induction

Half of the rats (*n* = 16) were injected intraperitoneally (i.p) with a single dose of streptozotocin (STZ), 60 mg/kg in a 2% solution of 0.1 M citrate buffer. In the Furman (2021) study is described that on the second day after the injection of a high dose of STZ, there is an increase in glycemia with values higher than the control which could reach superior 300 mg/dL values. However, there may still be remnants of pancreatic activity, representing the initial stage of DM1 [16]. In the present study, the experiments were started after one week after streptozotocin-induced diabetes. DM was confirmed by polydipsia, polyuria, and glucose levels greater than 300 mg/dL [17, 18]. The control group (*n* = 16) was administered saline i.p with the same volume. This procedure did not influence the glycemia of the control rats observed in the control group.

### Preparation of noni juice in natural

Noni fruit was obtained from Colatina City, Espírito Santo State in Brazil. Mature noni was washed; the seeds were removed; and the pulp was put into a blender set on pulse mode. The pulp (in grams) was passed through an extra-fine sieve and diluted in 0.5 mL water at a ratio of 2:1.

The bromatological analysis and bioactive compounds of the noni juice were carried out by a commercial laboratory (Hidrocepe Serviços de Qualidade Ltda). The bioactive compounds, phenolics, vitamin C, and flavonoids were quantified by enzymatic gravimetric, gas chromatography, and fluorescence methods, according to industry standards.

### Familiarization protocols

After arriving at the laboratory, all rats were introduced to a treadmill designed for small rodents (Modular Treadmill, Columbus Instruments, OH, USA). The familiarization protocol consisted of running on the treadmill for five consecutive days. The rats were encouraged to run by being given light electrical stimulation (0.5 mA, 0.5 mV) from a grid at the rear of the treadmill belt. Each daily session consisted of running at a constant speed (10 m⋅min^-1^) at an inclination of 5% for 5 min. Over the familiarization days, the speed increased gradually and ended at 15 m⋅min^-1^. This procedure was designed to teach the rats to run and avoid excessive stress during the tests [19].

In the same period, the rats were also familiarized with gavage, the technique chosen for the noni juice administration. This technique guarantees the ingestion of the correct amount established. This familiarization is also done to avoid stressing the animals during noni juice administration.

### Experimental protocol

After the familiarization protocols, the rats were submitted to the incremental workload running test (IWT) until fatigued to measure three performance indexes: maximal oxygen consumption (VO_2max_), time to fatigue, and workload as performance indexes. Workload was calculated as body weight × exercise intensity × exercise time × treadmill inclination [20]. The workload tests began at a speed of 10 m⋅min^-1^ (5% inclination) with increments of 1 m⋅min^-1^ every 3 min until fatigue [21, 22]. Fatigue was defined as the point when the animals were no longer able to keep pace with the treadmill for 10 s [23]. From the result of this test, the rats were divided into four balanced groups to guarantee the homogeneity of the metabolic rate among the groups at the beginning of the study. Two of these groups received STZ i.p for DM induction, while the other two were control groups. The DM group was also treated with special-acting insulin (Humulin NPH^®^, São Paulo, SP): injections of two international units (UI) in the morning at 8:00 and other two UI in the evening at 6:00 (pilot data) to reduce mortality due to high levels of blood glucose. Control animals received the same volume of isotonic saline solution s.c for the same condition between groups to be guaranteed. Every three days, capillary glycemia was measured by a drop of blood formed from a small cut performed at the end of the tail. Glycemia was measured through the enzymatic analysis with the glucometer (Accu-Check Performa®; Roche Diabetes Care Brasil LTDA, São Paulo, Brazil). This glucometer uses an electrochemical method containing glucose oxidase.

Twenty-four hours after DM confirmation, all rats were submitted to the second IWT to verify its effects on performance and VO_2 max_. Following each group was divided into rats that received noni juice or a placebo (water) administered by gavage. Noni juice or placebo was administrated at a dose of 2 mL/kg once a day at 9:00 a.m., for 60 days. The groups were denominated as control + placebo (CP); control + noni (CN); DM + placebo (DMP); DM + noni (DMN). The dose was based on in previous study by Nayak et al. (2011) that observed a significant reduction in glycemia with 2 ml/kg of the noni juice.

**Fig. 1:**
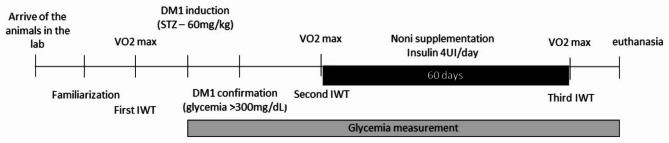
Overview of the experimental protocol design

After the noni administration period, all groups were submitted to a third IWT to verify the effect of noni juice on exercise performance and glycemia. All four groups performed the IWT between 2:00 and 5:00 p.m. to prevent circadian interference on performance or metabolism. After 24 h, all animals were euthanized (Fig. 1).

### Euthanasia

Six hours before the euthanasia, chow was removed from the cages, and the animals were left to fast. At approximately 8:00 a.m., the animals were euthanized by decapitation. The blood from the trunk was collected for fasting analysis and the tissues were removed. Kidney samples from euthanized rats were obtained and processed for histopathological evaluation.

### Histopathological analysis

Kidney samples were fixed in 10% buffered formalin for 24 h and embedded in paraffin for tissue sectioning (5 μm thickness). The sections were stained with hematoxylin and eosin (H&E) and evaluated under a microscope (BX53, Olympus Latin America Inc.) adapted to a microcamera (Q-Color3, Olympus Latin America Inc., SP, Brazil).

For the histopathological analysis measurements, the H&E sections images were digitized using a color video camera attached to a microscope. After digitalization, Bowman’s capsule and the glomerular tuft were traced and their areas were calculated using image analysis software (ImageJ). The area of ​​Bowman’s space was determined by the difference between the area of the glomerular tuft and Bowman’s capsule [24]. Fifty glomeruli were measured in each histological sample from each animal. The H&E sections were examined and scored by an observer in a blinded manner for the described parameters.

### Statistical analysis

The data were reported as mean ± SEM. The normality and homoscedasticity of data distribution were verified using the Ryan–Joiner and Levene test. The differences among the groups were evaluated by one-way analysis of variance (ANOVA). To evaluate groups and time points, a two-way analysis of variance (ANOVA) followed by Student–Newman–Keuls tests was conducted. The effect size (ES), measured on the Cohen’s d scale was considered for the analysis of data having a coefficient of variation above 30%. ES values were considered trivial (< 0.2), small (0.2–0.5), medium (0.5–0.8), or large (≥ 0.8). Correlations were assessed using Pearson’s coefficient.

All statistical analyses were performed using the SigmaPlot software (version 11.0; SYSTAT software, Bangalore, Karnataka, India), adopting a significance level of α = 5% (p < 0.05).

## Results

### Results of bromatological analysis

The results of the bromatological and bioactive compound analyses of noni juice are demonstrated in Table 1. Data indicated the presence of antioxidant and anti-inflammatory compounds such as flavonoids (0.017 g/100 mL), phenolic compounds (0.95 g/100 mL), and vitamin C (50.989 mg/100 g).

**Table 1:** Bromatological and bioactive compounds analysis of Noni (*Morinda citrifolia L*) juice *in natura*l

Parameter	Value
*Carbohydrate*	0.38 g/100 mL
Food Fiber	3.63 g/100 mL
Total lipids	< 0.18 g/100 mL
Proteins	0.40 g/100 mL
Calories	3.12 kcal/100 mL
Phenolic compounds	0.95 g/100 mL
Flavonoids	0.017 g/100 mL
Sodium (Na)	92.70 mg/L
Vitamin B2	< 0.06 mg/Kg
Vitamin C	50.989 mg/100 g

### IWT until fatigue before DM induction

Body weight, performance, and metabolic indexes were measured during the first IWT. Values indicated that body weight was not different among groups (*p* = 0.70). In addition, time to fatigue, maximal velocity, workload, and maximal oxygen consumption did not differ among the groups (*p* > 0.05 for all indexes; Table 2). Data indicated that groups were divided in a balanced way, with neither variable had shown differences among them (Table 2).

**Table 2:** Incremental workload test until fatigue before diabetes induction

1° IWT	CP (*n* = 8)	CN (*n* = 8)	DMP (*n* = 8)	DMN (*n* = 8)	*p* value
Body weight (g)	238.8 ± 5.2	229.8 ± 7.7	242.7 ± 6.6	241.1 ± 12.9	0.70
Time to fatigue (min)	53.0 ± 4.9	55.8 ± 4.6	49.1 ± 5.3	53.8 ± 4.3	0.77
Maximal velocity (m.min^-1^)	27.2 ± 1.6	28.3 ± 1.4	26.0 ± 1.7	27.8 ± 1.3	0.72
Workload (kgm)	32.1 ± 4.8	32.9 ± 3.7	28.8 ± 4.3	32.4 ± 3.4	0.87
VO_2max_ (mLO_2_.kg^-1^ min^-1^)	64.7 ± 3.2	67.0 ± 2.3	64.3 ± 4.0	67.5 ± 2.8	0.85

Data were expressed as mean ± SEM. Significant differences were considered if p < 0.05

### Effect of DM induction on IWT

**Fig. 2:**
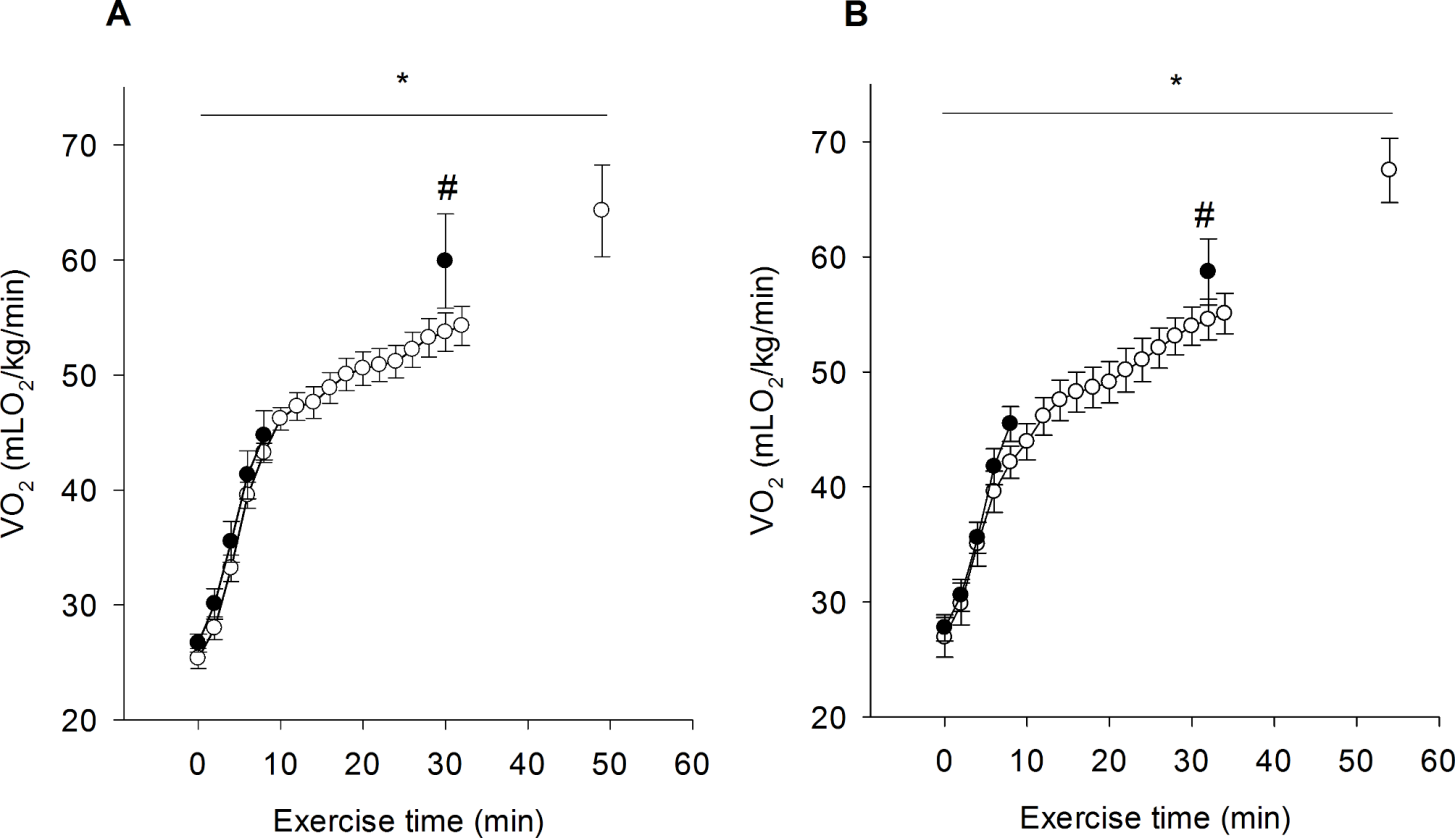
Effect of diabetes induction on exercise time and VO_2max_ in DMP (Panel A) and DMN (Panel B) rats. White and black symbols represent the values measured in 1° IWT (before DM induction) and 2° IWT (after DM induction), respectively. Data were expressed as mean ± SEM. Significant differences were considered if p < 0.05. * represents a difference from the beginning of the exercise. (*n* = 8 in each group). # represents a difference between groups

The IWT induced a progressive increase in VO_2_ in both DMP and DMN groups from the beginning of the exercise (*p* < 0.001). As illustrated in Fig. 2, DM induced a marked decrease in VO_2max_ in both groups DMP (64.3 ± 4.0 °C, 1° IWT vs. 59.9 ± 4.1 °C, 2° IWT; *p* < 0.001, Fig. 2A) and DMN (67.5 ± 2.8 °C, 1º IWT vs. 58.7 ± 2.9 °C, 2° IWT; *p* < 0.001, Fig. 2B). In addition, DM induction reduced by 39% the time to fatigue from the first IWT to the second in the DMP and DMN groups (Figs. 2A and 2B*p* < 0.001).

### Effect of chronic noni juice ingestion on performance indexes

As a result of the third IWT, the performance of the control groups CP and CN was lower performance than that of the second. This result may have been related to the weight gain and growth of the rats since a body mass of approximately 400 g was observed. Thus, to compensate for the body mass effect, the workload of the animals was calculated (Table 3).

**Table 3:** Workload calculated during IWT before and after noni (*Morinda citrifolia L*) administration period (60 days after)

	DMP	DMN
Workload (kgm) before	16.3 ± 4.2	18.2 ± 5.2
Workload (kgm) after	17.1 ± 2.3	24.6 ± 4.8
Variation (before and after)	0.7 ± 3.0	6.4 ± 2.8
Cohen’s d (effect size)	0.07	0.45

DMP: Rats with diabetes that were supplemented with placebo. DMN: Rats with diabetes that were supplemented with noni juice. Data were expressed as mean ± SEM. Significant differences were considered if p < 0.05. Cohen’s d (effect size) was considered small 0.20–0.30, medium 0.40–0.70, big > 0.80

**Fig. 3:**
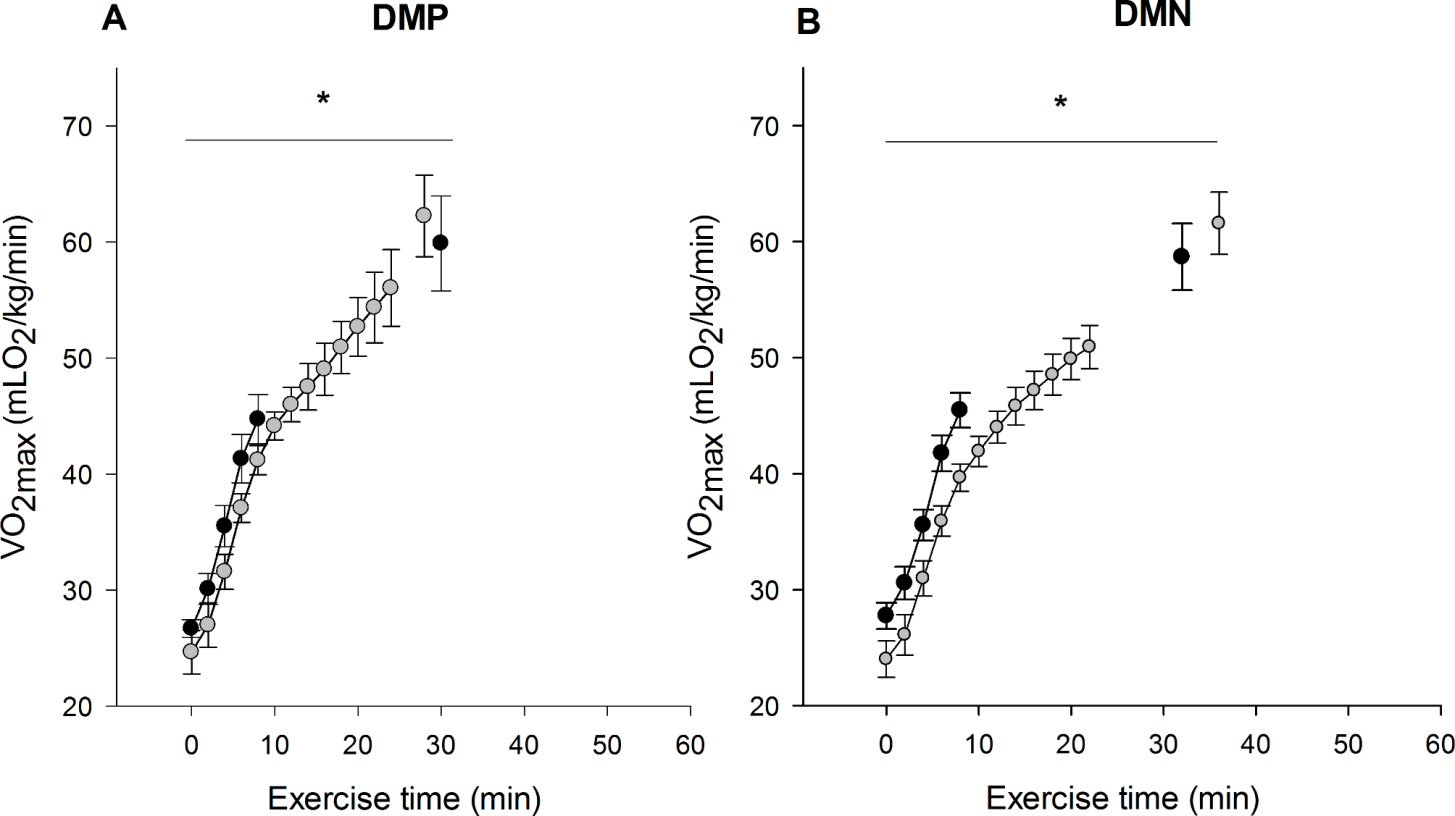
Effect of chronic noni (*Morinda citrifolia L*) administration on exercise time until fatigue and *VO*_*2max*_ in diabetic rats. Black and gray symbols represent the values measured in the IWT test before and after the 60 days of noni administration, respectively. DMP: Rats with diabetes that were supplemented with placebo. DMN: Rats with diabetes that were supplemented with noni juice. Data were expressed as mean ± SEM. Significant differences were considered if p < 0.05. * represents a difference from the beginning of the exercise. (*n* = 8 in each group)

The DMN group recorded the highest values for the third IWT after noni ingestion compared to the second (Table 3). This improved performance could be also observed through the exercise time (Fig. 3B) and exercise time variation between after and before noni ingestion (DMP − 2.1 ± 5.0 min vs. DMN 2.6 ± 3.6 min; *p* = 0.04, Cohen d = 0.53).

**Fig. 4:**
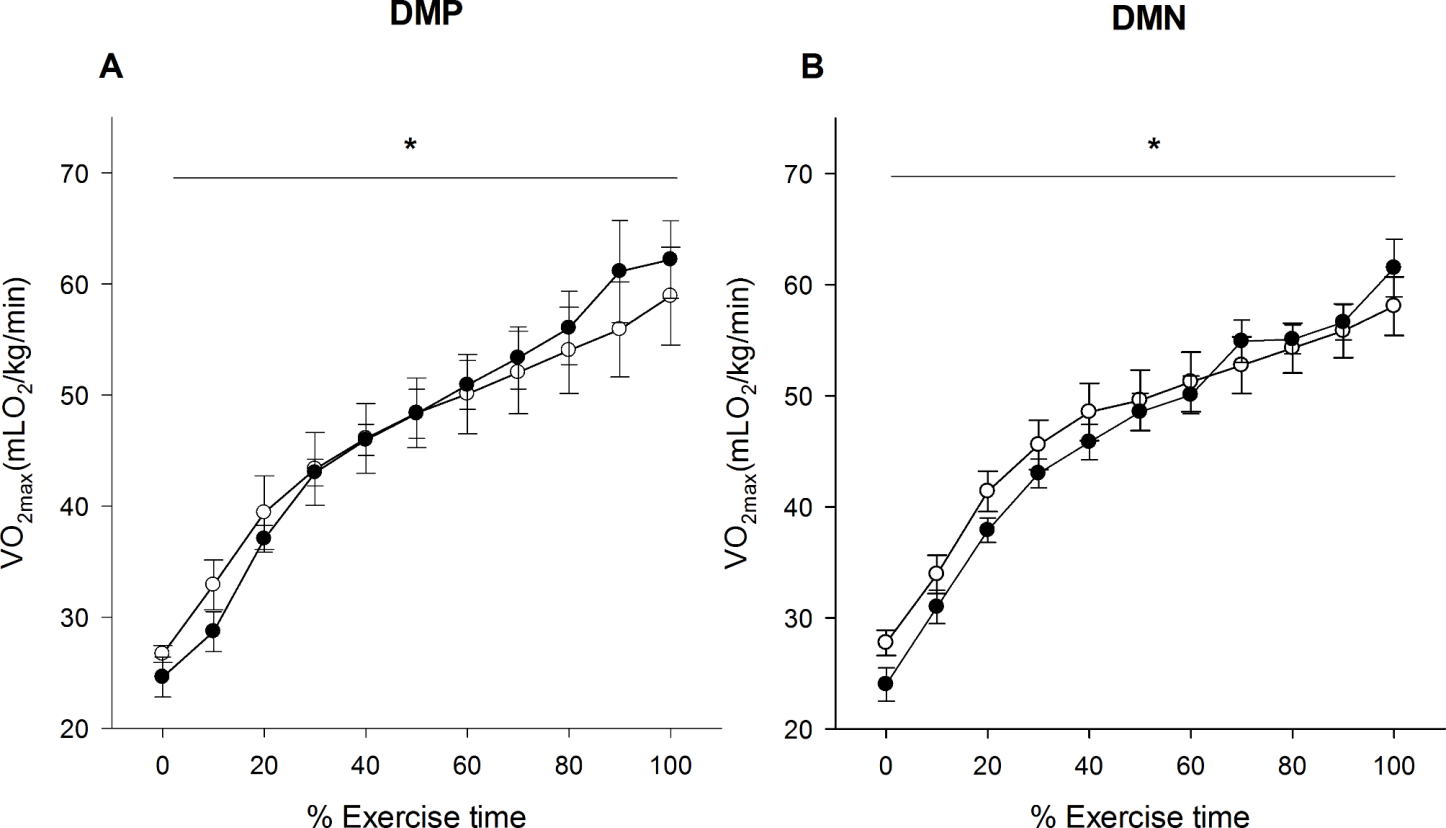
Effect of chronic noni (*Morinda citrifolia L*) administration on exercise time until fatigue and *VO*_*2 max*_ in diabetic rats. Black and gray symbols represent the values measured in IWT test before and after the 60 days of the noni administration, respectively. DMP: Rats with diabetes that were supplemented with placebo. DMN: Rats with diabetes that were supplemented with noni juice. Data were expressed as mean ± SEM. Significant differences were considered if p < 0.05. * represents a difference from the beginning of the exercise. (*n* = 8 in each group)

Since performance parameters improved after chronic noni administration, the energetic efficiency was analyzed by oxygen consumption as a function of the exercise time percentage. As illustrated in Fig. 4, oxygen consumption between groups DMP and DMN showed no difference, indicating that noni increased energy efficiency over the same time of exercise.

### Effect of chronic noni juice ingestion on glucose and triglycerides blood concentration

**Fig. 5:**
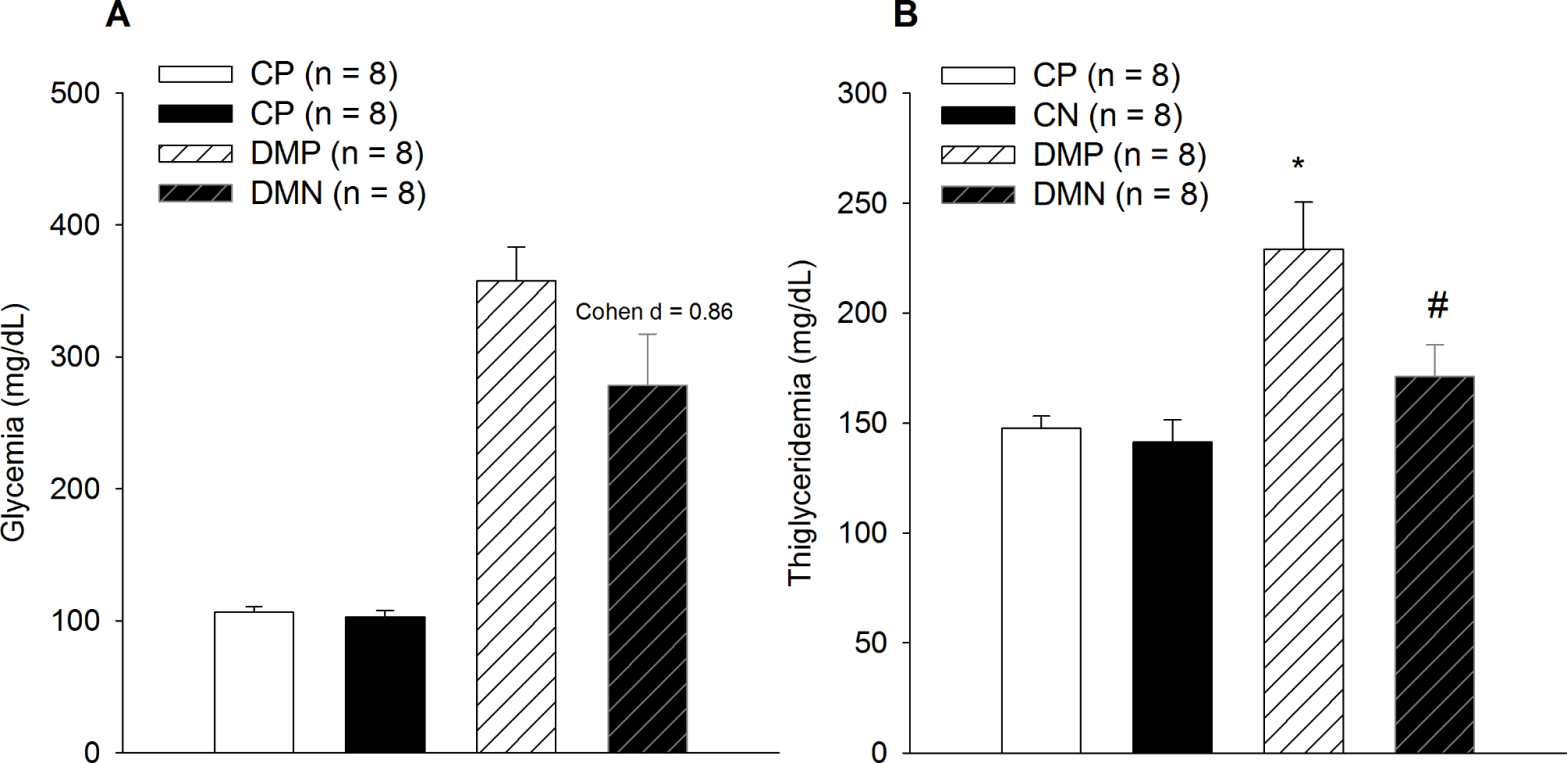
Effect of chronic noni (*Morinda citrifolia L*) administration on glycemia (panel A) and triglyceridemia (panel B). Data were expressed as mean ± SEM. Significant differences were considered if p < 0.05. * represents the difference from CP. # represents a difference from the DMP group

Chronic noni administration reduced the mean blood glucose calculated by the values obtained during the 60 days (large effect size Cohen’s d = 0.86, Fig. 5A). This result may imply improved glycated hemoglobin values (DMC 13.2% vs. DMN 10.9%).

The diabetic rats showed increased triglyceridemia compared to the control group (CP 147.6 ± 5.5 mg/dL vs. DMP 228.9 ± 21.5 mg/dL, *p* < 0.001, Fig. 5B). Triglyceride concentrations were determined by enzymatic colorimetric assays (Lab Test® Diagnostic S.A., Lagoa Santa, Brazil). After diabetic rats were supplemented with noni triglycerides, the values returned to the control condition and were lowered compared to those of the DMP group (DMP 228.9 ± 21.5 mg/dL vs. DMN 171.2 ± 14.2 mg/dL, *p* = 0.04).

From triglycerides and glucose concentration was calculated TYG index. The TYG index was calculated according to the following formula [Ln (fasting triglycerides (mg/dl) X fasting glucose (mg/dl)]/2; Ln is the neperian logarithm [25, 26]. The TYG index of DMP was higher than CP (CP 8.95 ± 0.06 vs. DMP 10.56 ± 0.16, *p* < 0.001). Rats that received noni supplementation attenuated the insulin resistance evaluated by TYG index, with a cutoff point of 7.88 was used for insulin resistance risk screening (DMP 10.56 ± 0.16 vs. DMN 9.97 ± 0.21, *p* = 0.04).

### Effect of chronic noni juice ingestion on kidney morphology

**Fig. 6:**
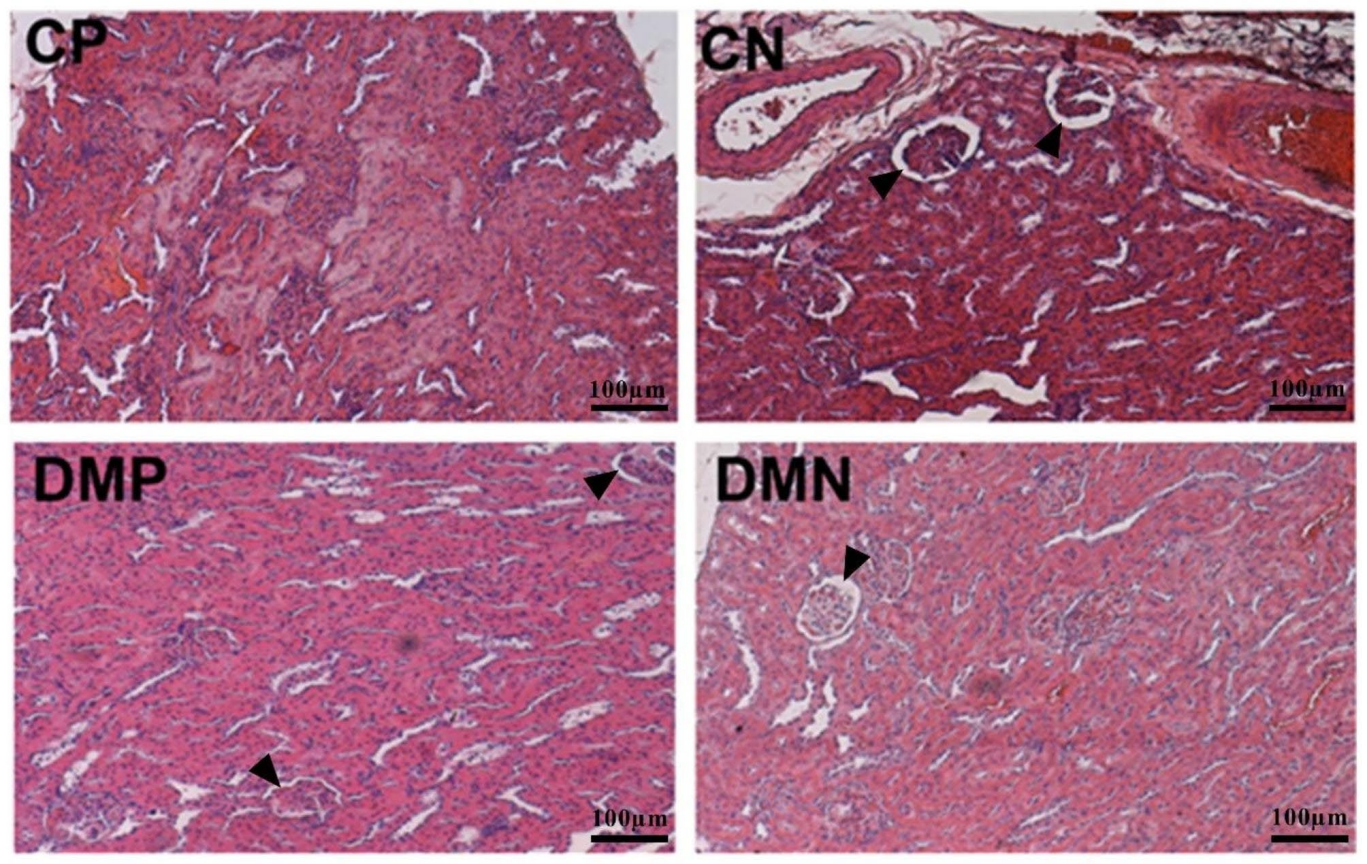
Photomicrography of kidney tissue of the control and diabetic rats after chronic placebo or noni (*Morinda citrifolia L*) administration. (CP: control + placebo; CN: control + noni; DMP: DM + placebo; DMN: DM + noni). Sections stained with H&E; Arrowhead indicates Bowman’s Space in groups with structural alterations. 10x increase in Microscope Nikon

In Fig. 6, noni ingestion produced an important increase in Bowman’s space area in the control rats, suggesting glomerular hyperfiltration (CP 0.60 ± 0.02 × 10^6^ µm^2^ vs. CN 1.48 ± 0.08 × 10^6^ µm^2^ ; *p* < 0.001). However, in diabetic rats, the noni supplementation did not produce additional impairment compared to CN (CN 1.48 ± 0.08 × 10^6^ µm^2^ vs. DMP 1.70 ± 0.02 × 10^6^ µm^2^ vs. DMN 1.98 ± 0.08 × 10^6^ µm^2^).

## Discussion

The present study showed that chronic noni administration in rats induced an improved mechanical efficiency associated with greater exercise time until fatigue. In addition, noni supplementation produced an antihyperglycemic effect and attenuated insulin resistance in DM rats. However, noni shows kidney toxicity through the increased Bowman’s Space, which can indicate their use is not safe in clinical or nutritional applications.

Comparable to our results, other studies have already demonstrated an ergogenic effect after noni administration [8, 27, 28]. Shalan et al. [8] observed that four weeks of noni supplementation tripled the swimming effort in rats. This increase in exercise performance was attributed to the peripheral and also to central effects induced by noni.

Centrally, our results demonstrated a reduced relative effort perception in DMN compared to the DMP group (Fig. 4). This fact could be evidenced by similar oxygen consumption values between groups when analyzed at the same relative performance, which was expressed as a percentage of running time. It is worth mentioning that both the time of exercise and workload (Table 3) were higher in the DMN rats. These data suggest a change in the central modulation that coordinates the motor drive and consequently induces delayed fatigue [29, 30].

Changes in the turnover of neuronal systems could play an important role in the peripheral adaptations associated with the development of fatigue. The Shalan et al. [8] study verified alterations to the central neurotransmitter systems, such as serotonin (5-HT) and dopamine (DA) receptors and transporters, associated with fatigue development in rats with noni supplementation. Furthermore, it has already been shown that peripheral signaling integrated with the central brain areas could modify the effort perception and consequently delay the end of the running time [31]. The neuronal 5-HT and DA system profile during exercise could change the running time until fatigue as a result of modifications to the lethargy, rating of perceived exertion, and motivation, which interfered with central brain signaling to the active musculature [32, 33]. It is important to point out that the experiment in this study was not designed to verify central fatigue. However, the observed peripheral effects could be attributed to both the drive from the central areas and feedback signaling changes.

Peripherally, the results of this study, such as increased energy efficiency in the DMN group (Fig. 4) could have been related to a direct action on central homeostasis neuromodulators, such as 5-HT and DA, contributing to energetic control to exercise but also a feedback from different muscle and metabolic conditions after noni supplementation. In addition, the DMN group showed a reduced hyperglycemic effect compared to the DMP group (Fig. 5). This result had already been reported by Osman et al. [28] and Shalan et al. [8] during swim exercise protocols and was thought to be the cause of an observed ergogenic effect. It is interesting to note that this study is the first to relate the effects of noni supplementation in rats with diabetes during controlled-intensity exercise performed according to a running protocol until fatigue.

Wang et al. [7] reported improvements in carbohydrate and lipid metabolism via the AMPK pathway in rats supplemented with noni. With specific regard to glucose metabolism, it had already been demonstrated an improved insulin receptor sensitivity beyond an increase in glycogen stores. It has been suggested that noni improves glycogen stores either by increasing glycogen storage, delaying glycogen consumption during exercise, or both [28]. These data contribute to blood glucose disappearing, probably through the improvement the carbohydrate muscle and liver uptake.

In the present study, the improved glucose metabolism in DMN rats could have been supported by a reduction in triglyceride plasma concentrations, which could indicate higher lipid oxidation (Fig. 5). This possibility is suggested by the reduction in the relative effort perception in the DMN group associated with a change in substrate use (Fig. 5). Data from Zhang et al. (2020) support that lipid metabolism was improved with noni supplementation accompanied by the increased expression of CPT-1 and PPARα, changing the fatty acid β oxidation [6].

In addition, some studies have associated the ergogenic and metabolic effects of noni administration with the influence of antioxidant compounds such as phenols and flavonoids (e.g., epicatechin and catechin) [7, 34, 35]. During exercise, there is an increase in the production of oxidative molecules, which may function as intracellular messengers in several physiological processes [36]. On the other hand, evidence shows that highly oxidative molecule concentrations represent possible toxicity and damage to the cell that could interfere with the excitation–contraction muscle process and consequently performance [37, 38]. The phenolic compounds and flavonoids present in noni have antioxidant and anti-inflammatory characteristics that may contribute to reducing the oxidative status promoting the effects observed on glucose and triglycerides metabolism, beyond the performance in DM rats with noni supplementation. Furthermore, it is suggested that the antioxidant and anti-inflammatory effects induced by noni supplementation may have contributed to the increased glucose uptake and consequent reduction in insulin resistance through pathways activation to GLUT4 translocation such as CA-calmodulin and AMPK [5]. In the present study, rats with noni supplementation exhibited reduced insulin resistance, calculated by the TYR index. However, this reduction did not reach similar values to the CP.

Although noni supplementation has increased performance and energy efficiency beyond having lowered glycemia and triglyceridemia, it appears to have a potentially toxicity effect on the kidneys as demonstrated by morphological analysis (Fig. 6). The increase in the Bowman’s space area in rats with noni supplementation (CN and DMN) suggested glomerular hyperfiltration. Our data also demonstrated an increase in Bowman’s space with the same magnitude of diabetic impairment in the control group that ingested noni. In the case of poorly controlled diabetes, there may be glycosuria and proteinuria as results of the deleterious inflammatory effect resulting from the reactive oxygen species formation, especially superoxide anion, which react with the glomerular matrix increasing the membrane permeability and consequent hyperfiltration.

Souza et al. [39] did not observe functional or histological disturbances in the kidneys or liver after nine days of noni juice consumption. Stands out that the juice dilution was lower compared to those mentioned in our study (1:10 for the Sousa et al. vs. 2:1 present study). In addition, the period of noni juice consumption in our study was extended (60 days), which may have contributed to the effect on the kidneys.

These impair the function of cycloxygenases and hydroelectrolytics, which then inhibit prostaglandin synthesis leading to chronic renal failure [40]. In addition, the potassium content present in noni can negatively impact kidney function in patients with renal failure (Mueller, 2000).

In addition, a other possible mechanism for kidney dysfunction is the hepatic changes induced by hepatotoxic components like as anthraquinones found in noni, which reduces anti-inflammatory molecules and initiate the lipid peroxidation [40]. In theory, noni could affect the kidneys from the liver since the organs are closely and are related in terms of metabolism and elimination of toxic substances.

However, it is crucial to understand that the effects of the bioactive compounds can vary depending on several factors, including dose, bioavailability and form of consumption.

## Conclusion

In the present study, chronic noni administration contributed to attenuating the hyperglycemia and avoiding the DM effects on performance during exercise until fatigue. However, the use of noni as a clinical and nutritional strategy in the treatment of DM must be understood with caution, since renal morphological change was observed.

## Limitation of the study

The results of the present study demonstrate glycemic and metabolic improvement with noni ingestion under conditions of experimental DM. This result cannot be directly transposed to humans. Thus further studies, including in humans, are needed for the use of noni as a nutritional strategy in glycemic control. Further, DM experimental induction does not promote similar whole organic conditions as compared to the natural disease induction, representing a limitation of the studies.

## Data Availability

The datasets analyzed in this study are available from the corresponding author (juliana.guimaraes@uemg.br) upon reasonable request.
